# Accidental Poisoning

**Published:** 1856-01

**Authors:** 


*Accidental Poisoning.*—A correspondent (Dr. J. H. Blake, of North Auburn, Me.,) mentions a case of poisoning by arsenic, which occurred lately in his practice, the mineral forming an ingredient of the coloring matter used for staining paper. A child was taken sick after chewing a green pasteboard show-card. An active emetic was administered immediately, and the boy was well the next day. On examination, it was found that the card was painted, or enameled, with a preparation of arsenic.—*ib.*

